# The Effects of Using Pancreases Obtained from Brain-Dead Donors for Clinical Islet Transplantation in Japan

**DOI:** 10.3390/jcm8091430

**Published:** 2019-09-10

**Authors:** Taihei Ito, Takashi Kenmochi, Kei Kurihara, Akihiro Kawai, Naohiro Aida, Yumi Akashi, Sakurako Kato

**Affiliations:** 1 Department of Transplantation and Regenerative Medicine, School of Medicine, Fujita Health University, Dengakugakubo 1-98, Kutsukakecho, Toyoake-shi, Aichi 470-1192, Japan; kenmochi@fujita-hu.ac.jp (T.K.); kurihara@fujita-hu.ac.jp (K.K.); ak.plus.kawai@gmail.com (A.K.); n-aida@fujita-hu.ac.jp (N.A.); 2 Transplantation Supporting Unit, Fujita Health University, School of Medicine, Dengakugakubo 1-98, Kutsukakecho, Toyoake-shi, Aichi 470-1192, Japan; akashi@fujita-hu.ac.jp (Y.A.); katosaku@fujita-hu.ac.jp (S.K.)

**Keywords:** islet transplantation, non-heart-beating donor, brain-dead donor

## Abstract

Background: The pool of brain-dead donors (BDDs) was increased with the revision to the relevant law in 2010, and islet transplantation from BDDs was started in 2013. The present study assessed the influence of using pancreases from BDDs on islet transplantation in Japan. Methods: The donor information registered with the secretariat of islet transplants from 2012 was reviewed, and the results of 86 clinical islet isolations performed in Japan between 2003 and 2018 with non-heart-beating donors (NHBDs) (*n* = 71) and BDDs (*n* = 15) were investigated. Results: The number of cases for which donor information was registered with the secretariat of islet transplants increased to 1.84 cases/month from 2013 to 2018 in comparison to 1.44/month in 2012, when only NHBDs were used. The median pancreatic islet yield was 275,550 IEQ (Islet equivalents) in the NHBD group but 362,700 in the BDD group, which amounted to a statistically significant difference (*p* = 0.02). As a result, 38/71 cases (53.5%) were achieved successful islet isolation (>5000 IEQ per recipient weight (kg)) was achieved in 38/71 cases (53.5%) in the NHBD group, and 12/15 cases (80.0%) in the BDD group; thus, the rate of successful islet transplantation was higher in the BDD group. Conclusion: The use of pancreases from BDDs has increased the overall number of cases for which donor information is registered with the secretariat of islet transplants and has improved the performance of islet isolation, thereby increasing the probability of successfully achieving islet transplantation.

## 1. Introduction

As shown in Table 1, which summarizes the history of pancreas/islet transplantation in Japan, clinical islet transplantation has been performed since in 2004 [1,2,3]; however, the long-term outcomes of clinical islet transplantation, as assessed by the Edmonton protocol, have remained poor in comparison to other countries. Anazawa et al. [4] summarized the outcomes of 18 recipients (8, 4, and 6 who received 1, 2, and 3 islet infusions, respectively), and reported that the rates of pancreatic islet graft survival at 1, 2, and 5 years were 72.2%, 44.4%, and 22.2%, respectively, whereas the corresponding graft survival rates after multiple infusions were 90.0%, 70.0%, and 30.0%, respectively.

From 2007 to 2011, islet transplantation was interrupted because of the potential for introducing transmissible spongiform encephalopathy (TSE) into islet cells when using collagenase [5]. New mammalian-free collagenase has been in use since 2011. However, according to several facilities, the results of islet transplantation have been significantly improved by induction therapy with a combination of anti-thymocyte globulin and etanercept as the induction therapy, according to several facilities [6,7,8]. Thus, a multicenter clinical trial of islet transplantation using anti-thymocyte globulin and etanercept for induction therapy was started in 2012 in order to improve the long-term graft survival in Japan, although the severe shortage of brain-dead donors (BDDs) in Japan presented another problem, as non-heart-beating donors (NHBDs) were the only viable source for islet transplantation. However, thanks to the revision of the relevant law in 2010, the BDD pool has gradually begun to increase, and islet transplantation from BDDs was started in 2013.

The present study evaluated the impact of using BDDs on the status of islet transplantation in Japan.

## 2. Materials and Methods

### 2.1. Transition of the Number of Brain-dead Donors (BDDs) and Pancreas Transplantation in Japan

To evaluate the introduction of the use of BDDs for islet transplantation, we compared the changes in the annual number of BDDs and pancreatic transplants before (2007–2009) and after (2010–2012), when the Japanese law related to brain-dead organ transplantation was revised. In addition, we evaluated the reasons as to why donor pancreases were not to be used for organ transplantation from 2010 to 2012.

All of data included in the analysis were provided by the Japan Organ Transplant Network, which is the only such agency in Japan.

### 2.2. Review of the Donor Information Given to the Secretariat of Islet Transplants

Islet transplantation from BDDs was started in 2013, and changes in the donor information sent to the secretariat of islet transplantation in Japan were examined. The monthly number of donors from 2012 to 2018 and the proportion of BDDs and NHBDs since 2013, when pancreases were first used for islet transplantation were examined.

### 2.3. Comparison of the Outcomes of Islets Isolated from Brain-dead Donors (BDDs) and Non-Heart-Beating Donors (NHBDs)

**Figure 1:**
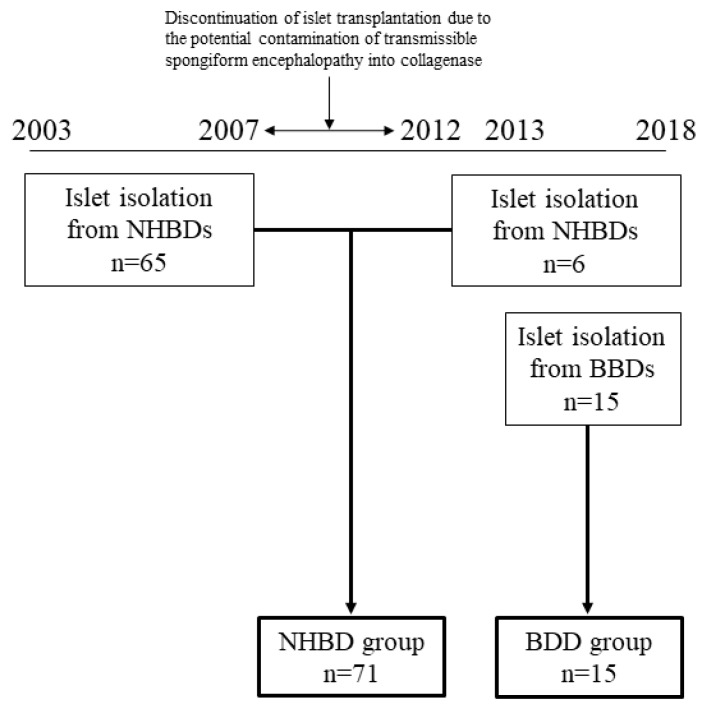
A flow chart of enrolled cases into the non-heart-beating donors (NHBD) and brain-dead donors (BDD) groups for comparison of islet isolation results. The islet isolation results donated from NHBDs in 2003–2007 and 2012–2018 have combined into the NHBD groups, while BDD groups have included the islet isolation results donated from BDDs only since 2003 when pancreases utilization has started for islet transplantation.

All data related to clinical islet isolation were registered and collected by the secretariat of islet transplants in Japan. The 86 clinical islet isolation procedures performed from 2003 to 2018 in Japan were divided into the NHBD group (*n* = 71) and BDD group (*n* = 15), and the donor characteristics and islet isolation results were compared between the groups (Figure 1). According to the Japanese criteria, islet isolation is considered successful when the islet yield is >5000 islet equivalents (IEQ) per weight (kg) of the recipient and islet transplantation is performed.

### 2.4. Statistical Analyses

All statistical analyses were performed using the EZR software program (free from the homepage of Saitama Medical Center Jichi Medical University), which extends the functions of R and R commander [9]. Fisher’s test was used to analyze categorical variables, and the Mann–Whitney U-test was used to analyze continuous variables. *p* values of <0.05 were considered to indicate statistical significance.

## 3. Ethical Aspects

All subjects gave their informed consent before they were registered with the secretariat of islet transplants in Japan, and information on the opt-out procedure was published on the Fujita Health University website (https://www.fujita-hu.ac.jp/). The study was conducted in accordance with the Declaration of Helsinki, and the protocol was approved by the Ethics Committee of Fujita Health University (HM19-030).”

## 4. Results

### 4.1. Review of the Transition of the Number of Brain-dead Donors (BDDs) and Pancreas Transplantation in Japan

**Figure 2:**
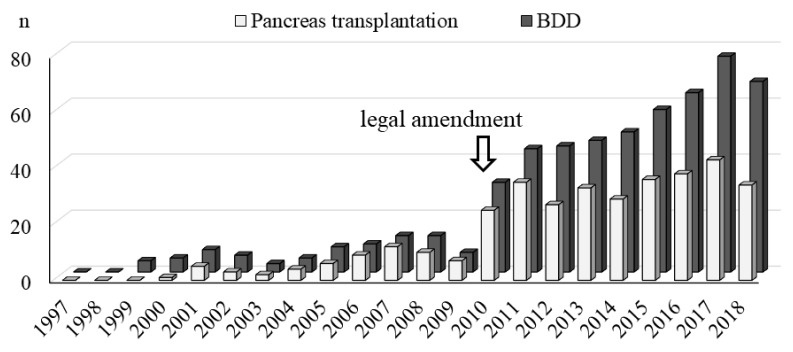
The annual number of brain-dead donors (BDDs) and pancreatic transplantation procedures. In Japan, the number of BDDs increased more than five-fold in 2010, after the revision of the law regarding organ donation by BDDs, and has gradually increased since then. The annual number of pancreas transplantation procedures has also increased in line with the number of BDDs.

The annual number of BDDs and pancreatic transplantations are shown in Figure 2. The number of BDDs, which was only a few cases a year prior to the revision of the law, has increased more than five-fold since 2010, when the revision of the law took effect, and has gradually increased since then.

**Figure 3:**
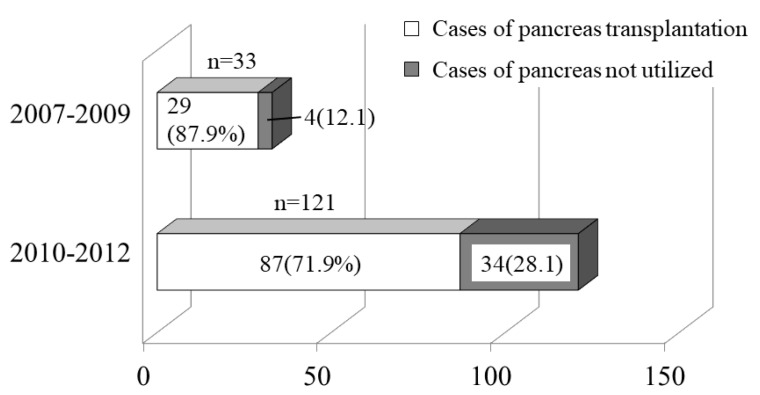
Use of pancreases procured from brain-dead donors (BDDs) for organ transplantation before and after the revision of the law. The percentage of pancreases procured from BDDs for organ transplantation was compared between 2007–2009 and 2010–2012. The percentage of pancreases procured from BDDs decreased from 87.9% before the revision to 71.9% after the revision.

We compared the numbers of BDDs and pancreases used for organ transplantation from 2007 to 2009 (before the legal amendment) and from 2010 to 2012 (after the legal amendment) (Figure 3). Transplantation from a BDD was performed in 29 (87.9%) of 33 pancreas transplants from 2007 to 2009, In contrast, transplantation from a BDD was performed in 87 (71.9%) of 121 pancreas transplants from 2010 to 2012.

**Figure 4:**
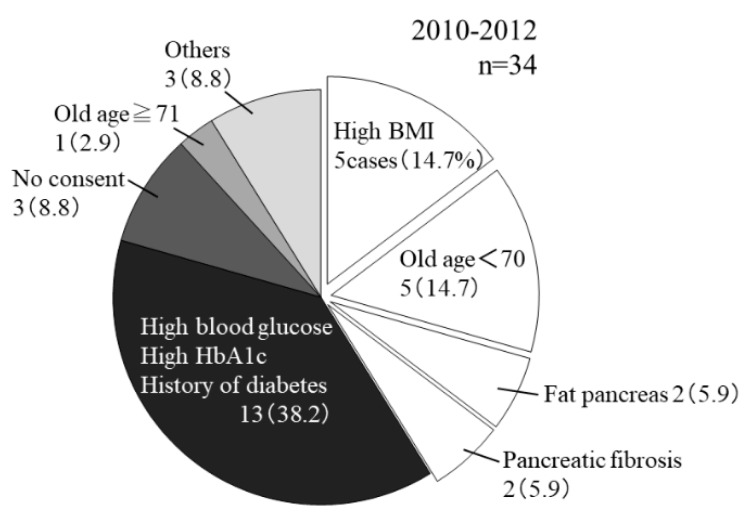
The analysis of the reasons for the non-utilization of pancreases from brain-dead donors (BDDs) for organ transplantation after the revision of relevant law. Pancreases from BDDs were not utilized for organ transplantation in 34 cases from 2010 to 2012. Depending on the situation, the possibility of diverting to islet transplantation was considered in cases in which the pancreas was not procured. The reasons for non-utilization of the pancreas in these cases included high body mass index (BMI), *n* = 5 (14.7%); age >70 years, *n* = 5 (14.7%); fatty pancreas, *n* = 2 (5.9%); and pancreatic fibrosis, *n* = 2 (5.9%).

We next examined the reasons why the rate of using pancreases from BDDs was clearly reduced from 2010 to 2012, we next examined the reason why (Figure 4). In 13 of 34 BDDs (38.2%), the pancreas was not procured for organ transplantation due to a history of diabetes, high HbA1c levels, or high blood glucose levels in the intensive-care unit, which suggested apparent glucose intolerance. However, in some cases in which pancreases were not used for organ transplantation, for reasons such as a high body mass index (BMI) and fatty pancreas, the pancreases were considered potentially useful for islet transplantation.

### 4.2. Impact of Brain-dead Donors (BDD) Utilization for Islet Transplantation on the Number of Donors

**Figure 5:**
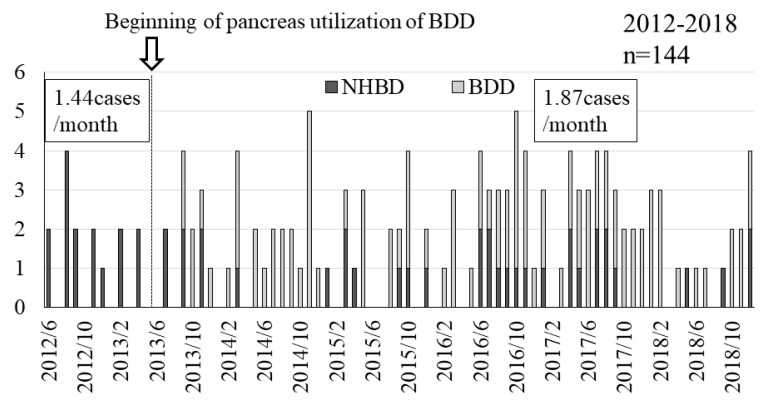
The number of islet donors whose information was given to the secretariat of islet transplants. From 2013 to the end of 2018, the number of donors whose information was given to the secretariat increased to 1.87 cases/month. In contrast, the number was 1.44/month in 2012, when only NHBDs were used; NHBDs, non-heart-beating donors; BDD, brain-dead donors.

**Figure 6:**
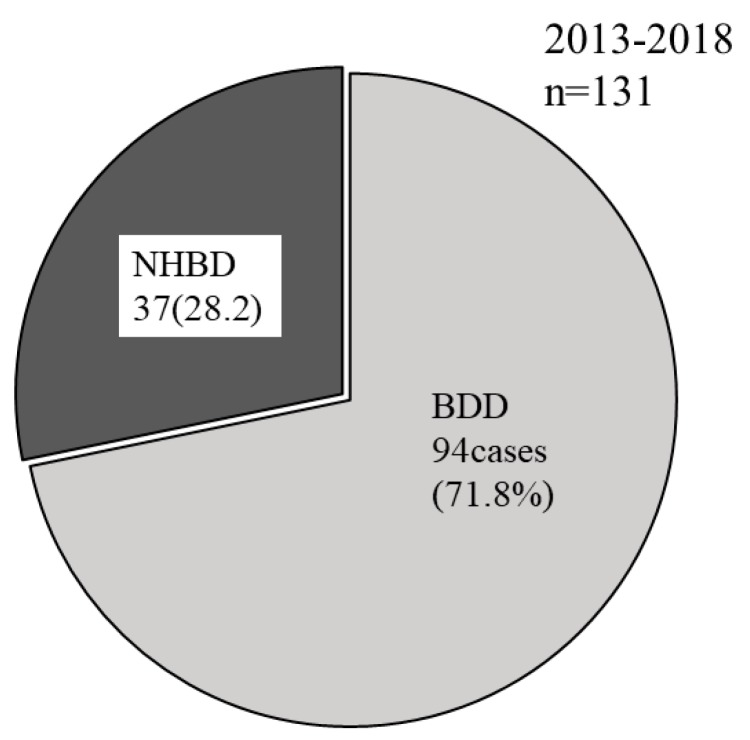
The proportions of registered non-heart-beating donors (NHBDs) and brain-dead donors (BDDs) from 2013. The utilization of pancreases procured from BDDs for islet transplantation was approved in 2013. By the end of 2018, BDDs accounted for 94 of 131 donors (71.8%) whose information was registered with the secretariat.

In 2012, when only NHBDs were used, the number of donors whose information was given to the secretariat was 1.44 cases/month. This increased to 1.87 cases/month from 2013, when BDDs became available for islet transplantation, to the end of 2018 (Figure 5). The utilization of pancreases procured from BDDs was approved for islet transplantation in 2013, after which the number of BDDs increased. At the end of 2018, BDDs accounted for 94 of the 131 donors (71.8%) whose information was registered with the secretariat (Figure 6).

### 4.3. A Comparison of the Donor Characteristics between Brain-dead Donors (BDDs) and Non-heart-beating Donors (NHBDs)

The donor characteristics are shown in Table 1. The donor age and sex ratios of the two groups were almost the same. Furthermore, there was no significant differences in the weight of the pancreatic grafts used for islet transplantation. However, in the BDD group, the median warm ischemic time (WIT) was much shorter, while the cold ischemic time (CIT) was significantly longer in comparison to the BDD group.

### 4.4. Comparison of the Outcomes of Islets Isolated from BDDs and NHBDs

**Figure 7:**
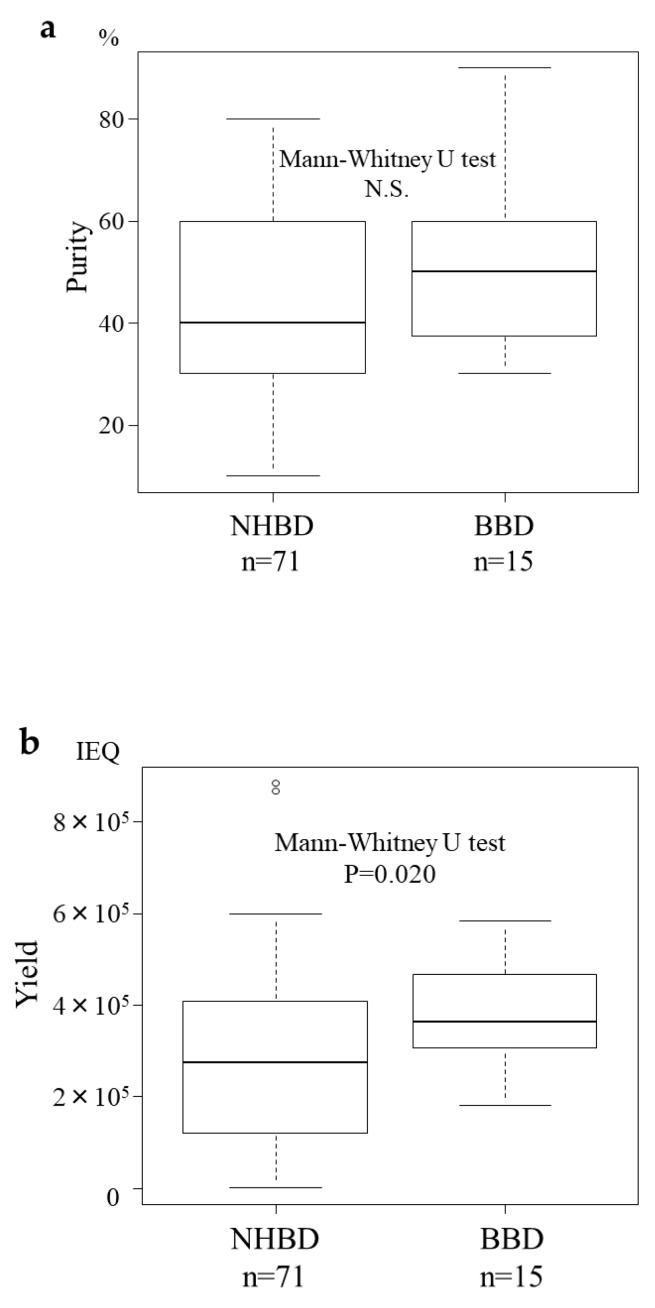
Comparison of the outcomes of islets isolatation from brain-dead donors (BDDs) and non-heart-beating donors (NHBDs). The median islet purity (**a**) and pancreatic islet yield (**b**). While the median purity after isolation did not differ to a statistically significant extent between the groups, the median pancreatic islet yield was 362,700 islet equivalents (IEQ 180,715–583,333) in the BDD group (*n* = 15) and 275,550 IEQ (400–885,608) in the NHBD group (*n* = 71), which amounted to a significant difference.

**Figure 8:**
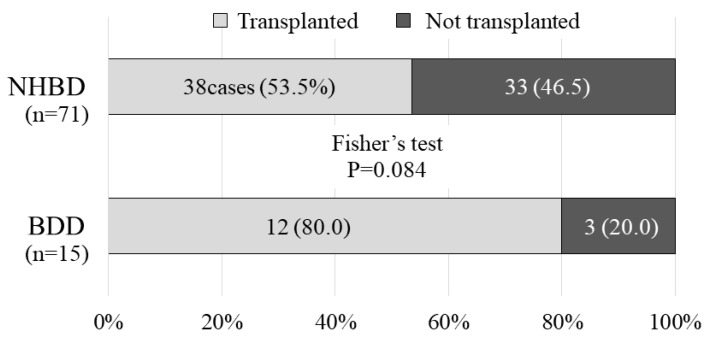
Rate of successful islet isolation in the non-heart-beating donors (NHBD) and brain-dead donors (BDD) groups. The rate of successful islet isolation was 12/15 cases (80.0%) in the BDD group amd 38/71 (53.5) in the NHBD group. Although the difference was not statistically significant (Fisher’s test, *p* = 0.084), the rate of successful islet isolation in the BDD group was higher than that in the NHBD group.

The median purity of islets after isolation was 40% (10–80%) in the NHBD group and 50% (30–90%) in the BDD group, and did not differ to a statistically significant extent (Figure 7a). However, the median pancreatic islet yield was 275,550 IEQ (400–885,608) in the NHBD group and 362,700 (180,715–583,333) in the BDD group, showing that the islet yield in the BDD group was significantly higher than that in the NHBD group (Mann–Whitney U-test, *p* = 0.020) (Figure 7b). As a result, 38/71 cases (53.5%) in the NHBD group and 12/15 (80.0) in the BDD group resulted in pancreatic islet transplantation. Although the result was not a statistically significant difference, the success rate of islet transplantation was higher in the BDD group than in the NHBD group (Fisher’s test, *p* = 0.084) (Figure 8).

## 5. Discussion

The revision of the law in relation to brain-dead organ transplantation has helped improve Japan’s serious donor shortage. As a result, islet transplantation using BDDs was started in Japan from 2013. The number of cases of pancreatic islet transplantation from BDDs has also increased, and improvements in islet isolation performance have resulted in an increased probability of achieving successful islet transplantation.

In Japan, we previously struggled to achieve a sufficient yield to transplant islets isolated from NHBDs [10]. Maruyama et al. [11] reported that the results of islet isolation were correlated with the kidney function after transplantation when both grafts were harvested from the same NHBD. It was revealed that because most NHBDs in Japan are “uncontrolled donors”, it is often necessary to perform dialysis after kidney transplantation, and the number of dialysis sessions and the serum creatinine level at one month after transplantation were correlated with the yield of islet isolated from the same donor as the kidney graft.

There are reports of islet isolation and transplantation from NHBD in countries other than Japan. Clayton et al. [12] reported no marked differences in the yield or insulin secretion between NHBDs and BDDs and no correlation between the islet yield and WIT in NHBDs. Markmann et al. [13] showed that the islet yield and function—assessed both in vitro and in vivo—were comparable between BDDs and NHBDs. In addition, they reported a case in which islet transplantation from a single NHBD successfully reversed type I diabetes. Zhao et al. [14] reported that up to 12.6% more islets were isolated from NHBDs than from BDDs with comparable viability. They further suggested that in cases with a WIT of ≤25 min, the islets from NHBDs were as suitable for clinical use as those from BDDs. In Japan, most NHBDs are uncontrolled and respirator-off is not possible. Thus, a substantial WIT, including an agonal stage longer than the WIT from the declaration of death to the start of cold perfusion, and the islet yield and function are likely to be highly affected by the agonal stage. However, the CIT in the BDD group was significantly longer than that in the NHBD group. The reason for this is because—in cases involving BDDs—pancreases are shipped all over Japan. In contrast, they are typically shipped only regionally in cases involving NHBDs, resulting in a shorter CIT.

One of the limitations of this study is that the impact of advances in the islet isolation technique and protocol cannot be ruled out. The NHBD group included the results of islet isolation from 2003 to 2018, while the BDD group only included the results of islet isolation from 2013 onward when pancreases were approved for utilization for islet transplantation. However, as shown in Figure 5, given the significant reduction in the number of NHBDs, the islet isolation results must be compared between these two groups. In addition, as shown in Table 2, the difference in the WIT between NHBDs and BDDs seems to have significantly influenced the islet yield, as the WIT in several NHBDs exceeded 15 min, and all of the islet yields in those cases were <300,000 IEQ, which is considered poor. Another limitation of this study is that the clinical trials are ongoing, and it is not possible to report on the progress of recipients after islet transplantation. Since there have been no major adverse events, and trials are ongoing, excellent outcomes are expected to be reported in the near future. Of note, a further analysis of the BDD group revealed that 2 out of 3 cases with an islet yield of <300,000 IEQ who were deemed unsuitable for transplantation due to such a low yield were ≥60 years of age. In other words, islet isolation for transplantation was successfully performed in 3/5 cases (60.0%) in cases involving BDDs of ≥60 years of age, and 9/10 cases (90.0%) involving BDDs of <60 years of age. Although an 80% success rate is still considered to be an excellent result for islet isolation from BDDs, the success rates are expected to be further improved through the stricter selection of donors as the number of BDDs in Japan continues to increase.

Prior to 2012, islet transplantation in Japan was only performed using pancreases from NHBDs; thus, there was no discussion about allocation between pancreas transplantation—which only used pancreases from BDDs—and islet transplantation. Since 2013, it has become possible to use pancreases donated by BDDs for islet transplantation. However, the pancreases that are used for this purpose were not used for pancreas transplantation due to reasons such as older age. In the near future, this study, which reports the utility of using pancreases from BDDs for islet transplantation, will play an important role in discussions about the allocation of pancreases from brain-dead donors between pancreas transplantation and islet transplantation in Japan. In addition, after the results of ongoing multicenter clinical trials are released, future studies will be able to compare the current results of pancreas transplantation in Japan and discuss allocation.

## 6. Conclusions

In conclusion, pancreas utilization from BDDs has increased the overall number of donors for islet transplantation whose information is registered with the secretariat of islet transplants. Improvements in islet isolation performance have resulted in an increased probability of achieving islet transplantation. In the future, islet transplantation from BDDs is expected to greatly contribute to the treatment of patients with Type 1 Diabetes in Japan.

## Figures and Tables

**Table 1:** The history of pancreas/islet transplantation in Japan.

1997	The enforcement of the law in terms of brain-dead organ transplantation in Japan “Islet transplantation working group” in the Japan Society for Pancreas and Islet Transplantation was established
1998	“Guidelines for islet transplantation” was issued
2000	Registration of patients for islet transplant recipient was started “The agreement for Islet Transplantation” was issued
2002	“Islet transplantation manual (first edition)” was issued
2003	The first Isolation of pancreatic islets from non-heart beating donor at National Sakura Hospital was performed
2004	The first clinical islet transplantation at Kyoto University was performed
2007	Islet transplantation was interrupted because of the potential contamination of transmissible spongiform encephalopathy (TSE) into collagenase
2010	The revision of the law in terms of brain-dead organ transplantation
2012	A Japanese multi-center trial of islet transplantation was started
2013	Utilization of pancreases donated from brain dead patients for islet transplantation was started

**Table 2:** Donor characteristics.

	Total	NHBD (*n* = 71)	BDD (*n* = 15)	*p* Value
Age (years)	45 (15–69)	45 (14–69)	46 (30–68)	0.571
Sex (male: female)	52:34	44:27	8:7	0.114
WIT (min)	3.0 (0–37)	4.0 (0.0–37.0)	0.0	<0.001
CIT (min)	314.5 (72–688)	305.0 (128.0–688.0)	411.0 (72.0–629.0)	0.046
Pancreatic graft weight	92.0 (37–183)	90.0 (37–183)	103.5 (72–173)	0.063

NHBD: Non-heart-beating donor, BDD: Brain-dead donor, WIT: Warm ischemic time, CIT: Cold ischemic time. Age was evaluated using Fisher’s test, and other items were evaluated using the Mann–Whitney U-test.
